# Editorial: Personalized radiation therapy: Guided with imaging technologies

**DOI:** 10.3389/fonc.2022.1078265

**Published:** 2022-12-06

**Authors:** Yingli Yang, Jing Cai, Davide Cusumano

**Affiliations:** ^1^ Department of Radiology, Ruijin Hospital, Shanghai Jiaotong Univeristy School of Medicine, Shanghai, China; ^2^ SJTU-Ruijing_UIH Institute For Medical Imaging Technology, Shanghai, China; ^3^ Department of Health Technology and Informatics, The Hong Kong Polytechnic University, Hung Hom, Hong Kong, Hong Kong SAR, China; ^4^ Mater Olbia Hospital, Olbia, Italy; ^5^ Fondazione Policlinico Universitario Agostino Gemelli IRCCS, Rome, Italy

**Keywords:** personalized radiotherapy, MR guided RT (MRgRT), biology guided radiotherapy (BgRT), functional imaging, image guidance

Over the years, with developments in technology and radiobiology, radiation therapy has evolved into a primary treatment method for many cancer patients with certain disease sites. However, in current radiotherapy (RT) practices, we are still treating each patient within a specific tumor type and stage with a common dose, ignoring the wide per-patient and per-tumor-sub-volume dose-response variations and missing the opportunity to dynamically modify the dose distribution based on tumor response (1). Radiation therapy (RT) efficacy is crippled by this lack of patient-specific treatment strategy. More and more studies have shown the value of personalized cancer treatments. Vendors and research institutes are also working on new treatment technologies with achieving personalized radiation therapy as one of the goals (2–5) Among all the newly introduced technologies, majority of them involves novel integration of imaging technologies. Such as, MR guided radiation therapy (MRgRT), biology-guided radiotherapy (BgRT) with onboard PET, CT guided RT, CBCT-guided RT with modern platform based on artificial intelligence (AI), and dedicated MR simulator for RT. In combination with the introduction of AI and radiomics into RT, online adaptive treatments and treatment response prediction are becoming practical to be included into clinical RT practice (6). The current issue highlights recent works in advancing personalized radiation therapy, specifically with the help of imaging guidance.

Among a number of proposals submitted, 19 of which were accepted for publication in the special issue. The accepted papers can be grouped in to the following three main directions: (1) importance of personalized radiation therapy; (2) image based treatment response prediction; (3) exploration of personalized treatment.

## Importance of personalized radiation therapy

Iezzi et al. presented a study on evaluating the dosimetric importance of on-line adaptive for breast IMRT treatment. A strategy is also proposed to make automatic prediction based on daily CBCT if on-line adaptive is necessary for that specific fraction. Also targeting on breast cancer, Wang et al. introduced their study on the incidental irradiation to internal mammary node (IMN) for patients underwent different type of surgery, radical mastectomy vs. breast-conserving surgery, and different radiotherapy regimens. Their study came to the conclusion that surgery type was the influencing factor of dose to IMN with conventional radiotherapy strategy. This opens up a question: is it possible to achieve more optimal dose to IMN regardless of the surgery type patients received with personalized RT? Zhang et al. used a “Sphere-mask” optical positioning system (S-M_OPS) retrospectively analyzed the setup errors for a large group of patients with different disease sites. In addition to introducing the efficiency and setup accuracy of S-M_OPS, the study also highlighted the residual setup errors with different mainstream setup tools, which can be further accounted for by on-line personalized RT.

## Image based treatment response prediction

Luo et al. introduced their model that is based on CT radiomics, clinical and dosimetric parameters to predict 1-year local control for lung cancer patients treated with SBRT. On the platform of low field MRgRT, Chiloiro et al. performed a study evaluating a “delta radiomics” approach to predict 2-year disease-free-survival (2yDFS) for rectal cancer patient undergoing neoadjuvant chemoradiotherapy (nCRT). For the same type of patients, Shi et al. investigated the usage of combined information of pretreatment blood biomarkers and MRI based morphological information to predict nCRT treatment response. Besides anatomical information, different medical imaging modalities can also provide functional information. Currently, anatomical change is still the main clinical criteria for treatment response evaluation. However, functional change, such as metabolism, cellular density, and vasculature, usually happens earlier than morphological changes (7–11) This is a highly desirable feature for treatment response prediction so that it can be used to improve treatment efficacy with early intervention. Zhou et al. performed a comprehensive review on the applications of functional imaging in liver-sparing RT. Kooreman et al. introduced an interesting study investigating longitudinal treatment response monitoring, using perfusion MRI techniques on a cohort of prostate patients. They evaluated two different perfusion MRI techniques, Intravoxel Incoherent Motion (IVIM) and Dynamic Contrast-Enhanced (DCE) MRI, finding significant correlations. This study highlighted the possibility of using IVIM as a non-contrast alternative perfusion MRI for longitudinal acquisition to achieve early treatment response prediction. For a cohort of head and neck patients, Chen et al. performed pre-treatment and weekly mid-treatment FDG-PET/CT acquisition during standard chemoradiotherapy. Tumor voxel dose-response matrix (DRM) constructed based on the serial FDG-PET/CT was proven to be a predictive tool for treatment response. Also with FDG-PET, Ji et al. developed a convolutional neural network (CNN) taking pre-treatment FDG-PET and spatial dose distribution as input to predict RT treatment outcome as a synthetic post-treatment FDG-PET, which can be used for adaptive RT decision making or on-line planning.

## Exploration of personalized treatment

Personalized treatment is a broad definition, and the personalized portion can happen at different steps of the entire RT workflow. Hooshangnejad et al. introduced a novel patient-specific duodenal pacer simulator algorithm, which can serve as a decision support system to provide optimal spacer location for placement guidance. Ku et al. introduced a novel fiducial marker (FM) implantation procedure by adding a patient specific pre-implant planning and simulation step. For patients with invisible lung tumors treated on CyberKnife, this retrospective study proved that the additional step reduces the patient radiation exposure and increases the number of FMs inserted around tumors. Zhang et al. explored an augmented reality (AR) – assisted RT positioning system using HoloLens 2. This is an interesting and novel patient specific positioning study and concluded that the proposed AR-assisted RT positioning method is highly feasible with several advantages. Using image guidance to personalize RT during treatment planning or treatment fractions is gaining a lot of research interests in the past several years. Zhu et al. used multiparametric MRI including 3D ASL to differentiate high and low blood perfusion areas within GTV for a group of adult non-enhancing low-grade gliomas (NE-LGGs). This generated information can be used to guide personalized RT boost for treatment efficacy improvements. Also for tumor segmentation purpose, Lau et al. explored a gradient-based method using 18F-PSMA-1007 PET/CT for prostate cancer lesion contouring and quantification. Nie and Li proposed predictive strategy to project tumor volume onto 2D MR cine from 4D MRI libraries for personalized MRgRT. By accurately predict respiratory motion during 2D cine imaging and projecting tumor volume contour on 2D cine, real-time assessment of beam-to-tumor conformality was proven to be feasible and promising for personalized MRgRT. Last but not the least, biology-guided radiation therapy (BgRT), represented by RefleXion X1™, the first FDA cleared BgRT system, is another novel and promising technology that can potentially bring meaningful personalized RT into routine clinical practice. Seyedin et al. described a planning comparison study on RefleXion X1™ and proved its potential as a powerful tool to reduce the radiation dose to nearby structures by using real-time positron emission imaging.

We have presented here some snapshots of different research activities in our field related personalized radiotherapy. We are hoping this can serve as a handy reference resource for students and researchers who are interested in this area and inspire more and more studies to further advance personalized RT with the ultimate goal of maximizing RT efficacy for every patient.
